# Transitioning Transdiagnostic CBT from Face-to-Face to Telephone Delivery During the Coronavirus Pandemic: A Case Study

**DOI:** 10.1177/15346501211018278

**Published:** 2021-12

**Authors:** Jess Saunders, Chris Allen

**Affiliations:** 1 Assistant Clinical Psychologist for Berkshire Healthcare NHS Foundation Trust, Berkshire, UK; 2 Consultant Clinical Psychologist for Berkshire Healthcare NHS Foundation Trust, Berkshire, UK

**Keywords:** COVID-19, CBT, modality, telephone, face-to-face, shielding

## Abstract

The coronavirus pandemic led to worldwide disruption in the delivery of face-to-face mental health services. This impact was marked for individuals with long-term health conditions and comorbid depression and anxiety. Many face-to-face mental health services switched to remote delivery or paused therapeutic input entirely, despite the lack of research on the efficacy of switching between modalities mid-therapy or having breaks in therapy. This paper presents the case of a patient with long-term health conditions who experienced both breaks in therapy and a switch in modalities from face-to-face to telephone delivery. The intervention used was based on transdiagnostic cognitive behavioral therapy and self-report measures were completed at the beginning and end of the twelve sessions. Despite the shift in modalities, the patient experienced clinically significant recovery on all measures, indicating the efficacy of therapy was not greatly affected by the shift in modalities. Long breaks in therapy were linked to deterioration in mental health, although this could be due to the deterioration in physical health that necessitated these breaks. This case highlights the benefits and challenges of a shifting modality of therapy during treatment and in response to a pandemic for a shielding population. From the work presented here, it seems beneficial for services to be able to work across multiple modalities to suit the needs of the patients and ensure continuity of treatment. It also indicates that pauses in therapy may risk deterioration. Further work is needed to prevent digital exclusion of patients.

## 1 Theoretical and Research Basis for Treatment

On the 23rd March 2020, the UK government announced a nationwide lockdown to combat the coronavirus disease 2019 (COVID-19). This had a dual impact on mental health services in the NHS. For the safety of practitioners in non-essential services, face-to-face services were cancelled, and certain patient groups who were high risk for COVID-19, such as older adults, and individuals with certain long-term health conditions (LTCs) (Garg et al., 2020), were asked to shield (Public Health England, 2020a). Shielding referred to limiting social contact by not leaving home or having face-to-face contact with others, unless necessary. This meant reduced access to non-emergency services.

In the general population, the stress of the coronavirus pandemic has been shown to provoke anxiety, stress, and low mood (Rajkumar, 2020). Yao et al. (2020) highlighted the expectation that the pandemic would exacerbate pre-existing mental health difficulties and Chevance et al. (2020) indicated that it could reduce access to services and support. The King’s Fund released a paper (Charles & Ewbank, 2021) highlighting the detrimental impact that the rapid changes in health service provision to meet the demands of COVID-19 have had on waiting lists and provision across services for the general public. Reports from the Office of National Statistics indicated an increased level of anxiety across the Great British public in May 2020 (Office for National Statistics, 2020). However, by the January 2021 report, mean anxiety had fallen, albeit not to pre-pandemic levels (Office for National Statistics, 2021). It is unclear how the pandemic will impact services and public mental and physical health long-term. It is vital that lessons are learnt from the first wave, that allow contingency planning for the future and to lessen the long-term impact.

Looking at shielding populations, specifically those with LTCs, the above research highlights a crisis in service delivery. Shielding individuals were socially isolated and those with LTCs have been shown to already be two to three times more likely than the general population to experience mental health difficulties over the course of their life (Naylor et al., 2012). Furthermore, comorbid mental health difficulties in this population have been linked with worsening physical health (Moussavi et al., 2007) and increased use of services (Egede, 2007). Therefore, a sudden cessation of previously face-to-face mental health support for these individuals poses a problem to both their physical and mental health. The UK government’s guidance on mental health and wellbeing during the coronavirus pandemic, emphasized the need for continuity in access of treatment where possible, suggesting arranging appointments with a therapist via text, telephone, or online (Public Health England, 2020b). However, to the best of our knowledge there is no evidence available on the effectiveness of switching therapy modalities mid-sessions.

Cognitive Behavioral Therapy (CBT) is one of the key interventions that the National Institute for Health and Care Excellence (NICE) guidelines suggest for adults with chronic physical health problems and either persistent subthreshold depressive symptoms or mild to moderate depression (National Institute for Health and Care Excellence [NICE], 2020). The guidance acknowledges both face-to-face, telephone, and computerized CBT as acceptable treatment options. Face-to-face (F2F) CBT has been shown to be effective for individuals with LTCs (Hynninen et al., 2010).

On 25 March 2020, Improving Access to Psychological Therapies (IAPT) UK published guidance on how to deliver treatment remotely during the pandemic (NHS England and NHS Improvement, 2020). This guide highlighted a relative lack of research on aspects of telephone delivery but concluded that existing research indicates that practiced therapists could achieve similar outcomes to F2F therapies. The general body of research does suggest that telephone-delivered CBT (t-CBT) is effective at reducing levels of depression for individuals with LTCs (Doyle et al., 2017), although the evidence for effectiveness on anxiety is less researched. Muller and Yardley (2011) conducted a systematic review of studies looking at t-CBT outcomes for individuals with chronic conditions and found these interventions improved their physical health outcomes. Therefore, t-CBT is an appropriate treatment for individuals with LTCs.

However, while there is evidence for the efficacy of both F2F and t-CBT for individuals with LTCs, there is little to find on the efficacy of switching between these modalities mid-therapy to respond to a crisis. It is important to examine whether a switch in modality impacts therapy now more than ever, due to the aforementioned stresses of COVID-19 and the possibility of exacerbation of pre-existing mental health conditions if service-delivery is adapted in an inefficient way. Holmes et al. (2020) highlighted the need for continuity in access to mental health support for vulnerable groups at this time. Considering the lack of research on the impact of significant breaks in therapeutic support on long-term outcomes for individuals with LTCs, it is important to ensure services continue and adaptations are effective.

Existing comparative studies are useful in raising potential strengths and weaknesses of each approach. For example, t-CBT has been highlighted as having lower attrition rates than F2F, (Mohr et al., 2012), providing visual anonymity (Lingley-Pottie & McGrath, 2007), having little impact on the formation of the therapeutic alliance (Irvine et al., 2020), and potentially being more satisfying for the patient (Simon et al., 2004). However, some have found t-CBT to be less effective long-term compared to F2F CBT (Mohr et al., 2012), disempowering for the clinician who is more reliant on the patient verbalizing and having access to the materials (Webb, 2014), more narrowly focused on CBT techniques (Webb, 2014), and vulnerable to issues with confidentiality and risk management (Brenes et al., 2012). Brenes et al. (2012) highlight these strengths and difficulties and suggest adaptations that a therapist could make to compensate for the different medium. This begs the question of whether CBT sessions that have a F2F and telephone component will benefit from the advantages of both mediums, suffer the disadvantages of both or be largely unaffected.

This case study examines the benefits, challenges, and effectiveness of transitioning modalities for an individual who was shielding. It will provide clarity on effective treatment options for higher-risk clients during a time of global pandemic and provoke discussion on how care can be adapted to meet present needs using the pre-existing research base available.

## 2 Case Introduction

AB was a 56-year-old Caucasian woman with the main presenting health conditions of Chronic Obstructive Pulmonary Disease (COPD), heart disease, and Type II Diabetes. Due to her health conditions, which made her high risk for COVID-19 complications, AB began shielding at the beginning of lockdown. AB was referred for mental health support via a multi-disciplinary team meeting, aimed at discussing complex cases. She was referred into this team meeting due to her number of health conditions which had left her housebound and her need for physical and mental health support.

## 3 Presenting Complaints

AB was seen by the Psychological Interventions in Nursing and Community (PINC) Service, which works within physical health services to deliver transdiagnostic CBT to patients identified in the community as having LTCs and comorbid anxiety and/or depression. AB had an assessment with the assistant clinical psychologist assigned to her case. This took place in her home. AB described her main concerns as being her low mood and anxiety. She suffered with chronic pain and fatigue which left her feeling like a burden when she was unable to provide practical support to her two children. She also reported having no pleasure in her daily activities and feeling highly anxious before leaving the house, often becoming physically sick. She was able to leave the house once a week, but this required a great deal of physical and emotional energy and she often needed family members to attend medical appointments with her. AB stated she had no thoughts of a suicidal or self-harming nature (PHQ question 9 = 0) but described passively feeling life was not worth living at times and spoke of a recent incident where she had deliberately harmed herself on impulse out of frustration. AB’s family was a strong protective factor and based on the assessment she was considered a low clinical risk for deliberate self-harm.

## 4 History

AB reported a history of emotional abuse and neglect as a child, eating disorder, and self-injury as a teenager, alcohol misuse as a young adult and hospitalization following a breakdown. She described how motherhood became a protective factor for her mental health at that time, although this became less protective once she began to experience long-term health conditions, as she felt these interfered with her ability to be the kind of mother she wanted to be and increased her perceived burdensomeness.

AB received a diagnosis of COPD when in her mid-30s and 5 years later, she was diagnosed with depression by her GP. AB had been taking daily Fluoxetine for 16 years, starting 2 years after her initial diagnosis of depression. She had frequent chest infections and eventually an acute exacerbation of COPD 14 years after her diagnosis. Her notes suggest that she had been housebound for at least 3 years before the commencement of PINC support and this appeared to be largely related to a foot injury, which has had an ongoing impact on her mobility and chronic pain. The most recent changes in her health prior to therapy had been a diagnosis of osteoporosis and type 2 diabetes mellitus when she was 55 years old. AB linked her low mood and anxiety closely to her long-term conditions and the impact these have on her daily life.

## 5 Assessment

At assessment AB scored 16 on the Patient Health Questionnaire (PHQ-9; Kroenke et al., 2001) which indicates moderately severe depression and a 12 on the Generalized Anxiety Disorder questionnaire (GAD-7; Spitzer et al., 2006) which indicates moderate anxiety. She also completed the Recovering Quality of Life Questionnaire (ReQoL-10; Keetharuth et al., 2018) and scored 18, which is within the clinical range.

## 6 Case Conceptualization

At supervision, post-assessment, it was agreed that AB met caseness for depression and anxiety alongside her COPD. As NICE guidelines recommend CBT in this instance, it was then discussed whether AB would be best placed within the PINC service or the IAPT long term health conditions pathway. Hadert’s (2013) systematic review highlighted some adaptations for how interventions could be made more effective for individuals with LTCs, including a recommendation to embed the psychological interventions for this group within physical services. Allen et al. (2015) piloted using trained practitioners of transdiagnostic CBT (Mohlman et al., 2008) within a physical health service, to deliver CBT to older adults with LTCs. This was found to be effective in reducing scores on scales of depression and anxiety and proved cost effective. Two case studies from the subsequently created PINC Service, have further indicated this efficacy (Slaughter & Allen, 2020; Walshe & Allen, 2020). As such it was felt that AB’s presentation of comorbid anxiety and depression with an LTC that left her housebound, would be best served by PINC, as the preference was to deliver face-to-face therapy in the client’s home. Once lockdown was announced, continuity of service delivery was felt to be better for the client than transferring her to the telephone based IAPT long-term health condition service.

**Figure 1.**
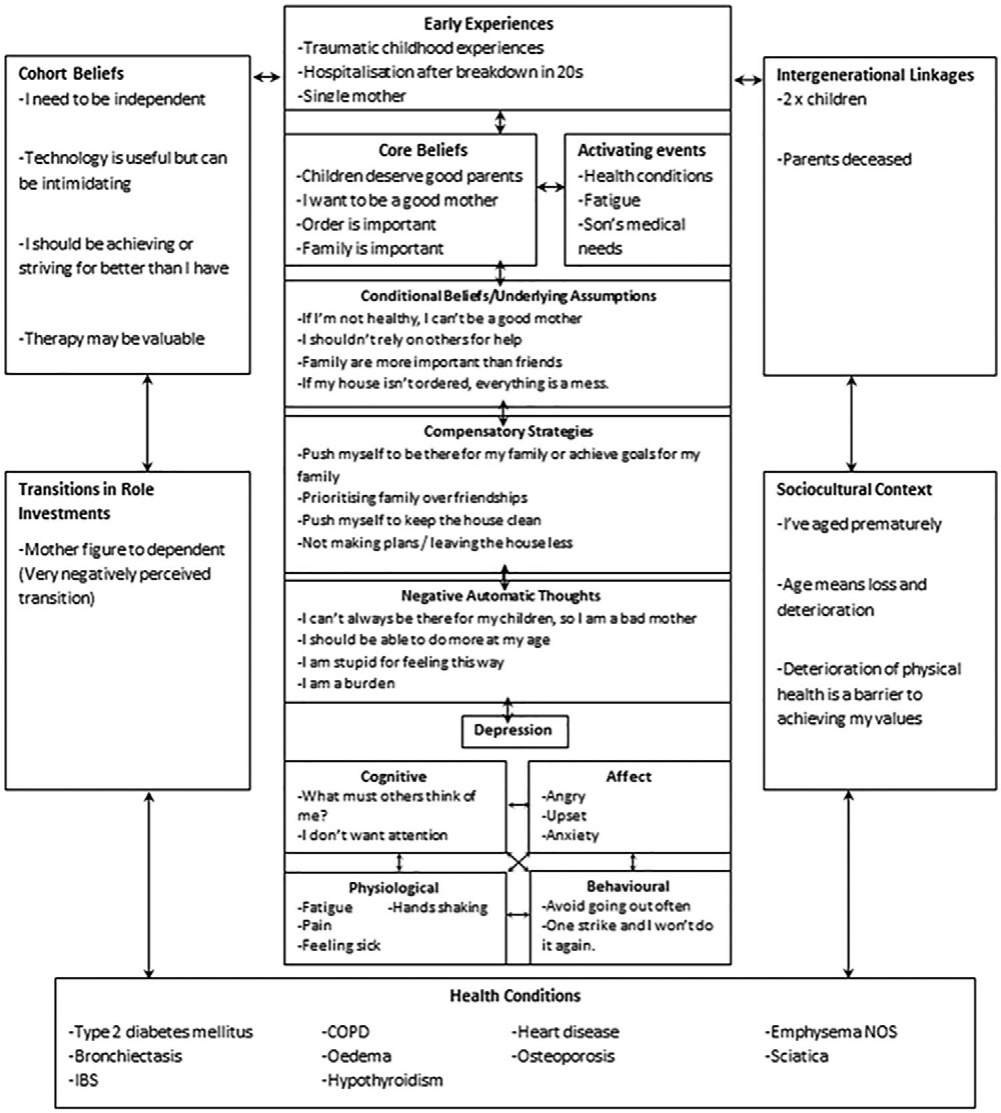
Laidlaw formulation for AB.

Figure 1 shows the formulation for AB’s treatment plan that was devised by her clinician over the assessment and first few sessions of therapy. This uses the Laidlaw Cognitive Behavioral Model for Older Adults (Laidlaw et al., 2004). While AB is not an older adult, this formulation is used within the service where this intervention took place. This is because the Laidlaw model captures the complex interplay of factors for individuals with both mental and physical health difficulties. This model also incorporates Padesky’s five aspects model of CBT (Padesky & Mooney, 1990), which was put together in session between AB and her therapist. The formulation highlighted AB’s anxiety about how she would be perceived both as a mother and as someone with an LTC, feeling a great sense of guilt and failure when her LTC or her anxiety prevented her from doing things she aspired to do. This, in turn, led to decreased motivation and more negative self-talk. The formulation also displayed her strong desire for independence and achievement, which motivated her to pursue therapy but at times led to rigidity in what progress should look like. On the basis of this formulation, it was agreed that it would be appropriate to start with psychoeducation around pacing for fatigue, anxiety management, and realistic goal setting, as well as introducing Acceptance and Commitment Therapy (ACT) ideas around values, to combat negative cognitions around being a failure when she was unable to achieve set goals.

## 7 Course of Treatment and Assessment of Progress

Sessions were planned to be delivered at the patient’s home by an assistant clinical psychologist trained in transdiagnostic CBT. Up to 12 sessions were offered, each around 1 hr long and offered on a one-to-one basis. The assistant clinical psychologist was supervised by a consultant clinical psychologist.

When lockdown measures were announced, patients seen by PINC were offered either to continue with CBT over the telephone or via online consultation, to transition to weekly telephone check-ins to monitor until they wanted to resume therapy, or to go back on the waiting list until face-to-face appointments were available again.

### Session 1: Psychoeducation and Goal Setting (F2F)

AB explained some of her current difficulties in more detail, which led into a discussion around what depression, anxiety, and CBT are. The therapist and AB went through a Padesky formulation using a difficult situation that had happened earlier in the week. This was used to practically illustrate the link between thoughts, feelings, behaviors, and physical sensations. AB then decided on two goals:

1) Feel more confident
2) Leave the house without/with less anxiety

The therapist and AB then worked together to get a better understanding of what it would look like for AB to feel more confident. AB felt that she would know she had become more confident if she could go to the shops by herself or go on a day trip.

### Session 2: Pacing and Anxiety Management (F2F)

The therapist explained the theory behind pacing for fatigue and suggested how AB could do this as well as demonstrating some anxiety management techniques, including relaxed abdominal breathing, progressive muscle relaxation, and grounding techniques. They discussed AB’s intention to go on a day trip. They broke down what the barriers to attending similar events have been in the past and agreed to explore how this task could be broken down in subsequent sessions. The therapist set AB homework of practicing relaxation techniques once per day and trying to incorporate pacing for fatigue into her schedule.

### Session 3-4: Values and Activity Selection (F2F)

AB had worked on pacing for fatigue and the relaxation techniques. AB had not found pacing for fatigue helpful but had problem solved ways of effectively completing activities while reducing fatigue. She was frustrated that, despite her progress, the coronavirus pandemic could prevent her from doing her day trip. AB and the therapist then went through a sheet asking about AB’s values and how successful she felt in each area currently. They then devised activities based around those values. The idea was to identify achievable activities that would enable her to live out her values. This idea was taken from Acceptance and Commitment Therapy (ACT; Hayes et al., 2012). Aspects of ACT have been indicated to be complementary to CBT for treatment of chronic pain (Lunde & Nordhus, 2009). She then rated each from 1 to 10 in terms of difficulty and then chose 10 activities from that list to put in a hierarchy, starting from the least difficult activity to the most difficult. The therapist and AB discussed moving through this list and AB agreed to try the first few that week. They also agreed on rewards for completion of every step of the hierarchy.

### Lockdown

After session 4, face-to-face appointments were cancelled for PINC due to the coronavirus pandemic lockdown. The therapist contacted AB and initially she felt that online consultations would be a suitable alternative for her. She was very positive about her progress and reported that she had not cried for a long time and was enjoying incorporating rewards into her schedule. AB was then ill with a chest infection for 5 weeks, leading to a break in therapy. Whenever AB had a stretch of illness, the therapist attempted to make contact on a weekly basis to determine when AB would like to resume support. On one such occasion, the therapist contacted AB’s GP to ensure that she was well, as AB had not been in contact. AB tended to be active in getting in contact with her therapist when she was happy for sessions to resume. AB generally did not continue with homework during periods of ill health.

### Telephone Check-In 1

AB’s anxiety around COVID-19 had increased and she reported finding the news coverage and the isolation due to shielding distressing. She reported a dip in mood as she had been crying intermittently throughout the week and felt an increased level of stress. The therapist reminded AB of the relaxation techniques and other strategies for coping with stress. AB requested to pause CBT in favour of weekly check-in phone calls.

### Telephone Check-In 2-3

AB was concerned about aspects of her health condition that had been deteriorating. She was reluctant to call a healthcare professional (HCP) about this due to fear of being exposed to COVID-19 through HCPs. AB felt her mood was generally good.

### Telephone Check-In 4-6

AB expressed increasing anxiety regarding COVID-19. She and the therapist discussed limiting her news intake and implementing a daily worry-time. The week after, AB had not done any worry time, but by check-in 6, she had incorporated this into her daily schedule. AB also described the days blurring together without distinction, so she and the therapist discussed activity planning to differentiate days. The therapist highlighted how thought challenging can be a helpful CBT technique and one they could go through together. AB agreed to resume CBT via video call the following week but was then unable to attend due to pain. The therapist decided to schedule in the next session over telephone as this medium had been working well.

### Session 5-6: Thinking Errors and Cognitive Restructuring (t-CBT)

The therapist and AB went through a list of different thinking errors. AB identified which errors she related to most and wrote examples of each from her own experience. The therapist set homework to try and note down five negative thoughts to be discussed in the next session. Table 1 shows one example of the cognitive restructuring she did. As homework, AB was given some blank cognitive restructuring worksheets to go through.

**Table 1.** Cognitive Restructuring Exercise.

Situation	Thinking about leaving the house after shielding
Feelings before restructuring	Concerned (50%)
Thought	“I’m going to have a panic attack when I leave the house after lockdown”
Evidence for	AB has had panic attacks in the past when leaving the house.
	AB had company when she was able to leave the house in the past. AB may not have anyone to go with her when she leaves the house next.
Evidence against	A family member said they can be available to help AB if she needs them.
	AB does not feel anxious when going to a familiar place.
	AB has left the house without panic attacks.
	Before lockdown, AB had stopped having panic attacks before leaving the house.
Alternative thought	“I could have a panic attack when I go out for the first time, but this is probably unlikely if I start with a familiar place and build up from there”
Feeling after restructuring	Concerned (30%)

### Session 7-9: Behavioral Activation and Avoidance Hierarchy (t-CBT)

The next session was one month later as AB had become unwell with a chest infection. AB expressed increased feelings of emptiness and a lack of pleasure in her daily routine. The therapist and AB discussed self-care and self-compassion explaining the theory behind behavioral activation. AB and the therapist worked together to make a behavioral activation schedule, reincorporating worry time, and did this in the subsequent session also until AB began to feel her mood had lifted.

The therapist and AB discussed her goals pre-COVID-19 and how they could be adapted. AB identified that her goal of feeling more confident could transfer from the day trip to attending a family celebration. The therapist and AB then worked together to determine what was making AB anxious about attending the celebration and what steps could be put together in an avoidance hierarchy to achieve this. AB agreed to try and do four steps before the next session but this was not achieved, partly due to illness. The therapist instead focused on a short worry exposure exercise, where AB visualized going to the event and identified the potential difficulties of this, allowing her to pre-empt some concerns with cognitive restructuring. The therapist also discussed cycles of avoidance with AB and self-compassion.

### Session 10-11: Sleep Hygiene, Worry Exposure and Positive Psychology (t-CBT)

There was another two-week gap between these two sessions due to AB being unwell. In this gap, the therapist sent over resources on sleep hygiene to discuss if needed. AB discussed attending the celebration but AB had not worked through the avoidance hierarchy and had found the event overwhelming. The therapist reinforced the psychoeducation behind the hierarchy and worked with AB on why building up to these things would lead to more positive experiences. AB had also stopped doing the anxiety management techniques, so these were reinforced. AB agreed to try creating her own exposure hierarchy for a new goal. It was agreed to review this next session. There was another two-week gap between session 10 and 11 due to ill health. In session 11, the therapist went through a worry exposure exercise with AB and positive psychology. AB chose to look at a positive psychology exercise for homework.

### Session 12: Relapse Prevention

The therapist and AB had a final recap of all the techniques they had covered and briefly explained mindfulness and signposted to resources. AB had completed the homework for that week. AB completed relapse prevention questions, aimed at identifying signs of setbacks and highlighting which techniques had been most helpful as well as future goals. AB then completed the outcome measures.

### Outcomes

The measures used pre- and post-intervention were the PHQ-9 to measure depression, the GAD-7 for anxiety, the ReQoL-10 for quality of life, and the Client Service Receipt Inventory (CSRI; Beecham & Knapp, 1992) to determine level of use of NHS services.

Pre-intervention, AB achieved a score of 16 on the PHQ-9, which is interpreted as moderately severe. This fell to a score of 8 post-intervention which is in the mild range. This is a clinically significant change as it is over 5 points and this also takes AB below the clinical threshold of 10 (Kroenke et al., 2010).

At the end of therapy, AB’s anxiety, as measured by the GAD-7, reduced from moderate (12) to within normal thresholds (4). This indicates a reliable change in score, in line with the index devised by Bischoff et al. (2020).

In terms of quality of life, at the start of therapy, AB’s ReQoL-10 score was in the clinical range with a score of 18 and by the end of therapy it was 27 which falls within the range of the general population. As this was also an increase of over 5 points this is classified as a reliable improvement.

**Figure 2.**
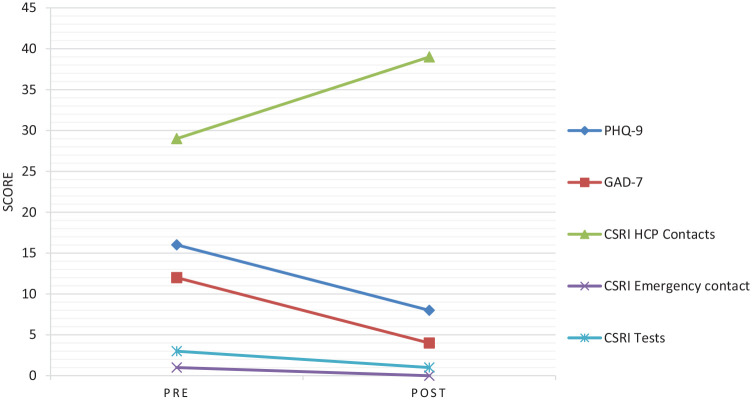
Change in score for outcome measures, pre- and post-therapy.

AB visited the GP, specialist doctors, and accident and emergency less over the course of therapy according to the CSRI. She saw the practice and specialist nurses, podiatrist and occupational therapist more frequently than at the start of therapy (Table 2). Some of these changes are insignificant, with visits from the GP, occupational therapist, specialist nurses and podiatrist only increasing by one or two visits. For the more significant changes, it should be noted that these scores will have been significantly disrupted by service changes during the coronavirus pandemic, as the data for the pre- and post-questionnaires ask the patient to think back over the last three months. For the pre-intervention questionnaire this would have gone to November 2019 and for the post-intervention it would have been July 2020. AB saw specialist doctors less, likely due to a need for greater urgency to access these services during lockdown, as many appointments were cancelled. The biggest increase was in social worker support, which again may have been aided by the accessibility of phone delivery versus F2F delivery. There was a shift from entirely face-to-face delivery to telephone delivery of services measured in the CSRI. Considering AB’s reported anxiety at assessment about travelling to clinics, this is likely to have been beneficial in facilitating contact with services and may explain increased contact with practice nurses. It is notable that many of these services were contacting AB, not the other way around. The increase in contact with certain services, such as practice nurses, may also be a positive indication. Throughout therapy, AB described being avoidant of medical appointments due to the high anxiety these appointments caused her. Her increased contact with HCPs may be indicative of worsening physical health symptoms or reduced anxiety, allowing more seeking of health support when needed. Figure 2 summarizes the results of the outcome measures for AB.

**Table 2.** Number of Contacts with HCPs Recorded in Client Service Receipt Inventory (CSRI) Questionnaires.

Contact with care provider	Number of contacts in the 3 months before therapy	Number of contacts in the 3 months prior to end of therapy	Difference between time 1 and 2
GP	6	5	−1
Practice nurse	2	5	3
Occupational therapist	2	3	1
Specialist nurse	0	2	2
Doctor other than GP for a physical health problem	6	0	−6
Podiatrist	9	10	1
Social worker	4	14	10

AB also set two goals at the start of therapy, to gain more confidence, as demonstrated by being able to go on a planned day trip, and to leave the house with less anxiety. The first goal had to be adapted due to the coronavirus pandemic. This goal was shifted to attending a celebration, which was achieved. Similarly, the second goal was paused during the shielding period but by the end of therapy, AB reported confidence in cognitive restructuring which reduced some of the physical sensations of anxiety she was feeling before leaving the house and enabled her to do so when needed. While the goals did need to be adapted, they were both met to AB’s satisfaction by the end of therapy.

## 8 Complicating Factors

Despite AB’s progress during the switch in modality, it is important to acknowledge that complicating factors were present to both AB and the therapist due to this switch. A key manifestation of this is in increased difficulty adhering to homework after the switch. While AB completed some of the homework, namely the behavioral activation schedule, the thoughts diary, and the positive psychology lists, there was significant difficulty with the avoidance hierarchy task and the continuation of activities like worry time and relaxation exercises. This is problematic as homework adherence has been associated with better outcomes (Mausbach et al., 2010). Haller and Watzke’s (2021) study indicated that reliance on homework is a notable feature of t-CBT, so lack of homework adherence does not appear to be an intrinsic disadvantage of t-CBT, but may instead indicate a potential flaw in the therapeutic approach. Haller and Watzke suggest that homework-related therapist behaviors are positively associated with homework engagement. These behaviors are listed as including clear, specific descriptions, having a cogent rationale, eliciting reactions from patients so difficulties can be troubleshooted, and summarizing progress at review. While none of these factors should be intrinsically affected by delivery method, it is possible that as service resources and the style of homework setting used by the service were based around F2F engagement, either therapist inexperience or an inadequate level of adaptation to the resources available impacted AB’s engagement with some tasks after a switch to t-CBT. For example, it is possible that using smart phone apps rather than emailing traditional worksheets, may have increased engagement.

Haller and Watzke’s study also indicated a decrease in homework engagement as therapy progressed, so it is also possible that the decrease in homework engagement was not a result of the change in modality but instead was impacted by the duration of therapy. It is also possible that this may just represent reluctance in the service user to engage in certain tasks. The avoidance hierarchy is anxiety provoking and the practice of worry time and relaxation techniques are effortful tasks; therefore, homework non-compliance could be unrelated to the delivery style and instead contingent on the type of task set. This idea is supported by AB’s reluctance to engage with the pacing homework at the start of therapy, instead adapting the homework to something she felt better suited her goals. Regardless, this highlighted a change in behavior after the switch, which may suggest there is greater difficulty engaging with certain tasks when switching to phone delivery.

Furthermore, the therapist also reported more difficulty doing and monitoring behavioral work over the phone. This is clear from the repeated revisiting of the avoidance hierarchy and the psychoeducation around it. This was also concerning from a risk management perspective, as the therapist being unable to monitor progress and reinforce homework, meant that the basis for making risk assessments was entirely on the information AB chose to report. Webb (2014) identified this as a disadvantage to remote therapies and something that is disempowering to the clinician. The therapist reported difficulty assessing how well AB understood various concepts and difficulty reinforcing this without handing worksheets directly to AB and helping her keep notes. Bateup et al. (2020) suggest that fidelity to the CBT model is a significant predictor of therapeutic outcome. The F2F sessions had focused on setting up behavioral goals, many of which could not be completed due to the pandemic, so resetting these goals led to some repeated work. The cognitive work on the other hand, worked well over the phone and was largely unchanged by phone delivery.

As can be expected with clients with LTCs, AB’s physical health frequently became a complicating factor in delivery of therapy. Both historically and during therapy, the link between AB’s physical and mental health was not a straight-forward bidirectional relationship. AB’s history of mental health difficulties began as a young adult, long before a diagnosis of COPD. However, AB disclosed that these mental health difficulties led to an eating disorder with physical health implications once again underlining the link between physical and mental health for AB. AB’s first diagnosis of depression from a GP was five years after her diagnosis with COPD but AB’s referral to the PINC service was not associated with a significant dip in physical health, which indicates a more complex picture. AB’s frequent illnesses during the course of therapy were not unusual looking at her medical notes, in fact, fewer records of physical illness were recorded in GP notes during the course of therapy than she had in previous years. The therapist noted that AB tended to self-report lower mood after a stretch of illness and AB clearly linked her anxiety and low mood to her LTCs at the start of treatment. The transdiagnostic approach utilized in treatment, considered locus of control, redirecting attention to what could be controlled through thought challenging and ACT. AB used worry time and noted at the end of therapy that she had found normalization, self-care, positive psychology, and relaxation techniques, particularly useful in managing her mental health alongside her physical health.

Similarly, it is notable that thought challenging in CBT is often used to challenge cognitions that may be overly negative, whereas negative cognitions around the pandemic may be appropriate. AB mentioned reasonable anxiety around catching COVID-19, as well as reintegrating with social events once shielding ended. The transdiagnostic CBT used already acknowledges this to some extent, as many of a client’s concerns regarding their health when they have an LTC are reasonable. As described above, the therapist utilized a mixture of ACT work and focus on what could be controlled, via worry time and activity selection to increase AB’s perceived locus of control. In the thought challenging work, the therapist encouraged AB to consider the specific anxieties around the pandemic, for example, that she would have a panic attack when leaving the house for the first time. They then worked to challenge this by drawing from past experience and focusing on previous strategies and resilience, despite the new situation faced by AB. By using a mixed approach of transdiagnostic CBT and ACT, as well as making minor adaptations to better acknowledge her justified concerns regarding the pandemic and LTCs, the therapist was able to help AB challenge her anxieties and facilitate a sense of control, without providing unrealistic reassurance.

## 9 Access and Barriers to Care

A move to telephone delivery meant sessions were still accessible. This was especially important for service users in shielding populations who were at risk of deteriorating mental health during social isolation. AB had some gaps in therapy due to health issues and the two longest gaps appeared to have had a detrimental impact on her mental health. The outcome measures at the end of therapy indicate the effectiveness of therapy for AB and that continuing therapy was more effective than postponing therapy until F2F was available again.

Despite the change in modality, sessions were also able to remain largely reminiscent of how they had been during F2F delivery. For example, email was utilized to deliver worksheets before and after sessions and this allowed AB to follow along with the CBT in a similar manner to how she had in F2F sessions. This presents a barrier when service users do not have access to email. However, worksheets can be sent via post in that instance. Not having access to technology, to support telephone therapy or as an alternative to telephone-based therapy (online), could produce a digital divide for those who are not technologically adept, such as older people and lower income groups who cannot afford hardware. The offer of technological support from staff has been found to be beneficial in supporting the switch from F2F to remote delivery (Banbury et al., 2019) and loaning equipment or facilitating access to equipment may also be necessary. AB was offered online therapeutic support but the therapist and AB agreed that telephone support appeared to be easier for AB to engage with, after multiple missed online sessions.

## 10 Follow-Up

A follow-up for AB was completed four months after discharge. This was planned for three months; however, AB was admitted to hospital at that time. The follow-up was structured around a general catch-up and re-administration of the outcome questionnaires. AB reported finding her hospital stay traumatizing and this had caused a dip in her mood and anxiety levels. This was confirmed by her slight increase in score in the PHQ9, where she scored 11 for moderate depression, GAD7 where she scored 8 for mild anxiety, and a decrease to 18 in the ReQoL which is within the clinical range again. AB continued to have difficulties with some areas of her social life but reported continuing techniques explored in therapy, such as pacing, daily goals, and relaxation techniques. Due to the dip in scores, it was agreed with AB that booster sessions would be appropriate, to reinforce some of the course material. This was planned to be carried out alongside access to the computerized CBT platform, SilverCloud, so AB would have full access to the course content to peruse when needed. As AB highlighted some social isolation difficulties, AB also agreed to a befriending referral.

## 11 Treatment Implications of the Case

AB’s improvement in all scores at the end of the intervention indicates that therapy had been significantly effective despite a switch in modalities. This aligns with research that indicates the effectiveness of both modalities separately. From looking at research on t-CBT or F2F CBT on adults with LTCs it would have been expected that these gains would be maintained (Doyle et al., 2017) but this was not the case at follow-up. It is important to note that this was influenced by a traumatic life event and may not be representative of the efficacy of the treatment. Furthermore, AB continued to use techniques she had accessed in therapy and she had maintained some recovery on the PHQ9 and GAD7, considering her scores at the start of therapy were both one interpretative band lower by follow-up. This indicates that clinicians can switch modalities in CBT delivery during treatment but follow-up may be necessary to reinforce progress.

Contrary to Webb’s (2014) findings, sessions also did not generally become more focused on CBT techniques after transition to t-CBT. Instead, there was a similar level of sharing on current circumstances as well as CBT techniques. It could be posited that this indicates that the nature of the therapeutic alliance established in the F2F CBT sessions was carried over to t-CBT, echoing the findings of Irvine et al. (2020).

While outcome measures were only taken at the beginning and end of therapy and follow-up, AB also self-reported on how she was feeling during therapy. It is notable that during the two largest gaps in contact, the five weeks between session 4 and the first telephone check-in and the month between sessions 6 and 7, AB reported a significant dip in her mood, the first time prompting the telephone check-ins and the second necessitating therapy to focus on behavioral activation work. Both breaks were caused by health conditions temporarily worsening, which may in turn have impacted mental health. Again, this pattern occurred in the follow-up, where lower mood and increased anxiety were noted after a dip in health. Equally, the breaks in contact could have been the issue. This appears to indicate that breaks in therapy are not advised where possible or that this kind of fluctuation is an inevitable challenge when working with individuals with deteriorating LTCs.

The six check-ins delivered appeared to maintain the patient’s wellbeing and by the end of the sixth session, both AB and the therapist felt it was time to transition back to full sessions. The best comparison for the check-ins in literature is provided by befriending or nondirected supportive therapy, which has been used as a comparative treatment to CBT in some research (Brenes et al., 2018; Doyle et al., 2017). Both Doyle et al. (2017) and Brenes et al. (2018) found that these alternative approaches lacked long-term effectiveness compared to t-CBT. As AB’s reported wellbeing did not appear to dip during the check-ins, this aligns with the above literature that suggests these alternative approaches do have a short-term effectiveness. It is notable though that some CBT techniques were taught during the check-ins, such as worry time. It is unclear whether this would have played a role in maintaining the patient’s wellbeing over and above befriending support. It would be interesting for further work to examine whether a hybridized check-in, largely containing befriending and active listening support but with some taught CBT elements, would be an efficient way of bolstering check-in support and whether the outcomes would be sustained. Nevertheless, these check-ins did appear to have some benefit in maintaining the client’s wellbeing, making them preferable to a break in therapy.

## 12 Recommendations to Clinicians and Students

This case highlights some of the benefits and challenges of shifting modalities mid-therapy. It presents the need for clinicians to reflect on ways to make their practice more effective in an unfamiliar modality but also supports the general premise that existing CBT practices are effective across different modalities during the course of treatment. It is also recommended, based on this case, that breaks in therapy should be avoided where possible and that some therapeutic involvement appears to be preferable to none. Future work could examine the impact of these breaks on a wider client group, as well as ways of bridging this gap when clients are unable to access therapy due to poor physical health. Check-ins that functioned similarly to befriending also appeared to be anecdotally beneficial in maintaining the client’s wellbeing, so it is worth considering how this too could be utilized while clients are waiting to receive therapeutic support and whether this could positively impact on outcomes.

Patient and therapist satisfaction were not recorded in this study and this could have been useful in assessing the impact of a shift in therapy. However, at the time of F2F delivery, a modality shift was not expected, and satisfaction measures are not routinely collected in the service. Future research could look into whether shifting modalities impacts satisfaction with therapy, as well as whether mixed approaches, establishing a rapport and delivering key sessions in person, but checking in on homework and utilizing relevant smartphone apps remotely, may be preferable for some clients. Delivered in an effective way, this kind of change to service delivery could facilitate a more flexible, patient-centered therapeutic approach, as well as giving greater caseload capacity to individual therapists. With an increasing focus on how therapies could be delivered remotely, it will be important that clinicians and students also consider ways to reduce the possibility of digital exclusion in client groups who may have difficulty accessing new technologies.

A focus on best practice in delivery of care across both typical and emergency scenarios is likely to become a key topic of discussion within the NHS and other care delivery systems across the world. In line with the wider debate regarding the long-term impact of the pandemic on the mental and physical health of the population, the effects of the pandemic will necessitate clinicians to continue to innovate.
